# Implementation Effect of a Pilot Urban Primary Health Care Model on the Usage of Health Care From Medically Trained Providers for Chronic Diseases Among Slum Populations in Bangladesh: Findings From an Implementation Research Study

**DOI:** 10.2196/76290

**Published:** 2026-05-29

**Authors:** Md. Golam Rabbani, Zakir Hossain, Khadija Islam Tisha, Kamrun Nahar, Zillur Rahman Sakin, Towhida Nasrin, Mohammad Wahid Ahmed, Md. Zahid Hasan, Chandrasegarar Soloman, Margub Aref Jahangir, Maya Vandenent, Shehrin Shaila Mahmood

**Affiliations:** 1 Health Economics and Financing, Health Systems and Population Studies Division, icddr,b (International Centre for Diarrhoeal Disease Research, Bangladesh) Dhaka Bangladesh; 2 UNICEF (United Nations International Children's Emergency Fund) Bangladesh Dhaka Bangladesh

**Keywords:** urban primary health care, Aalo Clinic model, chronic disease, health care usage, medically trained provider, slum population, Bangladesh

## Abstract

**Background:**

Ensuring affordable and quality primary health care for urban populations remains a persistent challenge in resource-constrained countries such as Bangladesh. The country’s fragmented and pluralistic urban health system exacerbates these challenges, making it particularly difficult to deliver primary health care to urban residents. In Bangladesh, the Aalo Clinic has been piloted to provide essential health services to low-income urban residents, including slum dwellers.

**Objective:**

This study aimed to assess how this model affects health care–seeking behavior from medically trained providers (MTPs) for managing chronic diseases.

**Methods:**

We conducted implementation research from October 2021 to August 2023 using a cross-sectional study design. To assess the effectiveness of the Aalo Clinic model, the baseline survey was conducted from January to February 2022, and the end-line surveys were carried out from April to June 2023 in the Korail, Mirpur, Shyampur, Dhalpur, and Tongi-Ershadnagar slums, which are adjacent to the clinics. In the baseline survey, we randomly selected 2000 households (with 9196 members), while 2033 households (with 8223 members) were interviewed in the end-line survey. We considered 8919 individuals in the baseline and 7994 individuals in the end-line, all aged 1 year or older. We used descriptive analysis and chi-squared tests to assess changes in health care usage from MTPs and applied logistic regression models to assess the model’s influence on health care usage from MTPs while controlling for other factors.

**Results:**

Among slum dwellers, awareness of Aalo Clinic increased by approximately 62 percentage points, from 11.46% (n=227) at baseline to 73.36% (n=1473) at end-line. The usage of health care for the management of chronic diseases from Aalo Clinics’ MTPs was significantly higher at the end-line period (n=63, 11.13%) compared to the baseline period (n=6, 0.59%). Regression analysis indicated that slum dwellers were approximately 23 times more likely (95% CI 8.77-61.80) to use health care from Aalo Clinic for chronic diseases in the end-line period compared to the baseline. However, there was no substantial change in health care usage from non-MTPs, which remained stable at around 38% (baseline: n=388; end-line: n=218) in both periods, and local drug stores continue to be the primary source for managing chronic diseases.

**Conclusions:**

Usage of Aalo Clinic MTPs for chronic disease care increased substantially compared to other MTPs among slum dwellers. However, reliance on non-MTPs, particularly local drug stores, remained stable. Ensuring the availability of essential medicines may further reduce dependence on non-MTPs among low-income urban slum residents. Future longitudinal studies are needed to evaluate the long-term impact on chronic disease management in Bangladesh.

## Introduction

In low- and middle-income countries (LMICs) such as Bangladesh, ensuring affordable and quality health care remains a persistent challenge, particularly in urban areas where the provision of basic health care is limited [1]. This challenge contributes to the global setback in making significant progress toward achieving universal health coverage (UHC) by 2030: a key objective of the sustainable development goals [2]. To address this issue of providing affordable and quality health care, the World Health Organization recommends strengthening health systems with a primary health care (PHC) approach, as it can significantly contribute to delivering essential health care services [3].

Bangladesh has achieved significant success in health, leading to reduced maternal and child mortality rates [4]. However, the pressing challenge now is to improve the health of impoverished urban populations. Rapid urbanization is underway, with large numbers of rural residents migrating to cities, driven by poverty, climate change, and the hope for better economic opportunities. Currently, approximately 40% of Bangladesh’s population resides in urban areas, with an estimated annual growth rate of 3% [5]. A significant portion of these urban residents live in informal settlements, such as slums, which have a concerning growth rate of 7% [6]. Cities with inadequate infrastructure are struggling to accommodate this continuous influx of people, resulting in impoverished slum dwellers facing challenges in accessing health care, among other difficulties. This situation highlights the weakness in the government’s health system implementation and the basic health care for slum dwellers [7,8].

The Ministry of Health and Family Welfare has the mandate for health across the country, but urban PHC is mostly delivered by the Ministry of Local Government and Rural Development Cooperation or the Local Governance Institutions, due to a decree providing them the authority to deliver PHC in urban areas [7,9]. However, with its limited capacity, it is currently unable to provide adequate PHC. As a result, access to quality PHC for urban populations, particularly vulnerable individuals such as slum dwellers, is limited. Evidence showed that fragmented health care delivery in urban areas has been caused by unclear roles, poor planning, and a lack of coordination between the 2 authorities, among other factors [8]. Consequently, untrained informal providers, for example, drug sellers and low-quality private clinics, are relied upon by underserved populations [7,8,10-12], which worsens the health of slum dwellers and leads to catastrophic health care expenditure [13,14].

Evidence shows that nongovernmental organizations (NGOs) and development partners have tackled problems in slums with innovative strategies [7,9,15-18]. To overcome the existing barriers to accessing health care, particularly essential high-quality PHC services for urban dwellers, an urban PHC model, namely the Aalo Clinic, is being piloted in selected urban low-income areas since 2021 [19]. We aimed to assess the implementational effect of the Aalo Clinic model in prompting health care usage from medically trained providers (MTPs) for chronic diseases and to identify factors that influence the health care–seeking behavior of the patients.

## Methods

### Description of Aalo Clinic model

With a view to providing accessible and affordable care to the urban poor, this model has been developed based on setting up a static clinic, namely the Aalo Clinic, and outreach services through “Dorgoray Shasthya Sheba”[20]. The key features of this model are shown in Textbox 1.

**Key features of the Aalo Clinic model.**

- Offers free medical treatment to city residents.
- Patients receive treatment for common, seasonal, and noncommunicable diseases, as well as Expanded Programme on Immunization vaccination and family planning services.
- Provides free medicines: a total of 23 different types of medications are currently being administered.
- Free 10 examinations are performed, including complete blood count, creatinine, erythrocyte count, dengue, and diabetes.
- Every step is digitized, from patient registration to prescription writing to test results.
- Once a patient has registered, clinicians can access information about their prior medical history for any future visits or follow-up care.
- The standard time for doctors to see a patient is 8-10 minutes.
- Operates 6 days a week, except Fridays and public holidays, in 2 shifts: morning shift from 8:30 AM to 2:30 PM and evening shift from 3 PM to 9 PM.
- Well-equipped (infrastructure, health workforce, or equipment) to provide primary health care services.
- Provides outreach health care services through mobile vans in the static Aalo Clinic areas, where patients can receive counseling services from paramedics, and if required, patients are referred to Aalo Clinics.
- Arranges awareness-raising activities for the respective community.
- Provides appropriate referral if required for more specialized care.
- Provides 25%-40% discounted diagnostic tests for patients referred from Aalo Clinics.
- Routinely monitors clinic performance for quality assurance.
- Records beneficiaries’ feedback to cater service according to their need.

In the Aalo Clinic model, services have been provided according to the essential service package. The currently available outpatient services at the Aalo Clinics and outreach points are listed in Textbox 2.

**Health care services offered by the Aalo Clinic model for the urban slum populations in Bangladesh.**

**Maternal, neonatal, child, and adolescent health care preconception, antenatal, delivery, and postnatal**

- Maternal and newborn care (antenatal care, postnatal care, and delivery referral, newborn care, etc)
- Child health and immunization (Integrated Management of Childhood Illness, acute respiratory infection, diarrhea, growth monitoring and promotion, Expanded Programme on Immunization, etc)
- Adolescent health (sexual and reproductive health, reproductive tract infection or sexually transmitted infection, dysmenorrhea, tetanus toxoid vaccine, nutrition, and counseling on mental health)
- Nutrition
- Child nutrition: assessment of nutrition status, prevention of malnutrition, and management of malnutrition
- Promote exclusive breastfeeding, complementary feeding, water, sanitation, and hygiene for children, and growth monitoring and promotion
- Support to moderate acute malnutrition children and refer severe acute malnutrition children
- Maternal nutrition (BMI screening of malnourished pregnant women, mid-upper arm circumference, and counseling for food)

**Noncommunicable diseases**

- Noncommunicable disease (hypertension, diabetes mellitus, chronic obstructive pulmonary disease, etc) consultation and referral
- Mental health consultation
- Sexual and gender-based violence

**Management of other common conditions**

- Eye care and referral
- Skin care and referral
- Geriatric (older adult) care and referral
- Minor infection and disease
- Health education and counseling

Family planning (method selection counseling, provide oral contraceptive pill, condom, injectables, and intrauterine device)

Communicable diseases (consultation on communicable diseases, referral, and consultation on sexually transmitted infection or sexually transmitted disease)

Laboratory urinalysis, Hb estimation, blood grouping and Rh typing, blood sugar, complete blood count, and electrocardiogram

**Figure 1:**
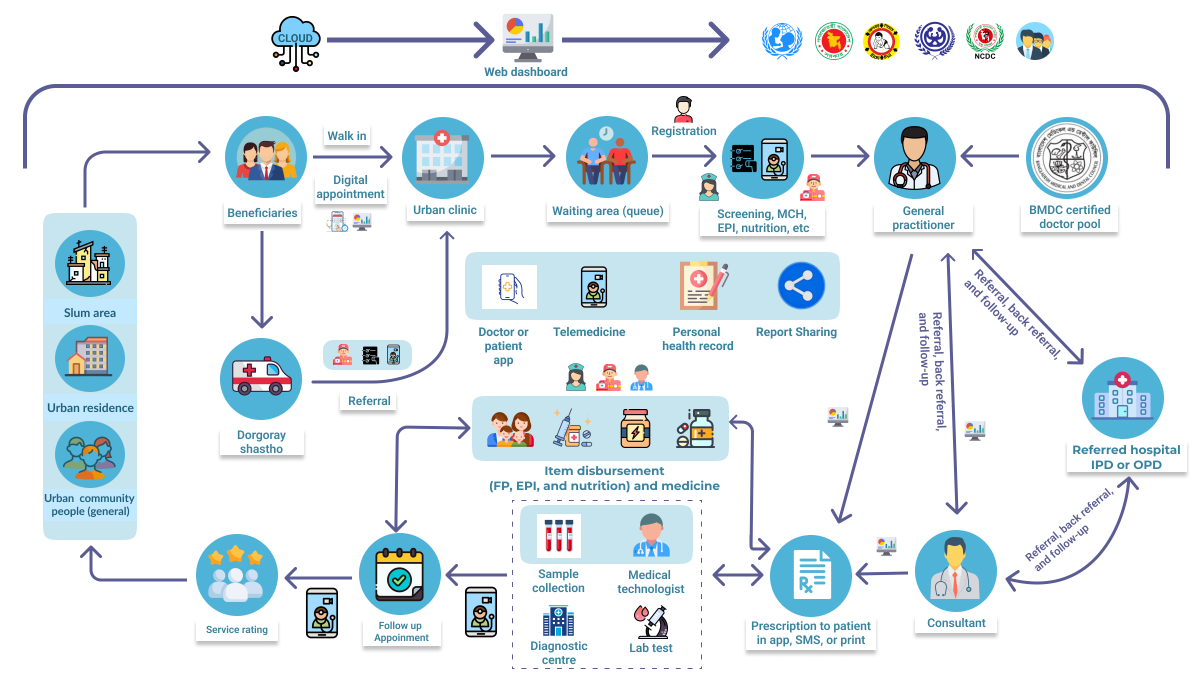
Service flow of the Aalo Clinic model and its information technology-based management system for health care delivery to urban slum populations in Bangladesh (adapted and reproduced with permission from UNICEF Bangladesh). UNICEF: United Nations International Children's Emergency Fund.

The health care services of Aalo Clinic are delivered in a structured way. During the first visit to the Aalo Clinic, a patient got registered on an IT-based system using their mobile phone number. Following that, the patient receives an automated serial number to get on the queue for general practitioners (GPs). After the screening by the GPs, the patient obtains free diagnostic tests and medicines depending on the prescriptions. The doctor can refer to a higher level of health care facilities if required. Figure 1 shows the overall service flow of the Aalo Clinics.

### Study Design

We conducted implementation research with the Aalo Clinic model between October 2021 and August 2023. With this study as a key component, we carried out repeated cross-sectional household surveys with selected slum dwellers, a primary focus group of this model, to assess the effects of the model on the usage of PHC for managing chronic diseases. The baseline survey was conducted from January to February 2022, shortly after the model’s service delivery began, while the end-line survey took place from April to June 2023, one year after its implementation. While both surveys targeted the same population group, the same households were not necessarily sampled at both time points.

### Study Setting and Population

The Aalo Clinic model has been implemented in 6 selected areas with Dhaka North City Corporation, Dhaka South City Corporation, Gazipur City Corporation, and Narayanganj City Corporation. In Dhaka North City Corporation, 2 Aalo Clinics were established and located adjacent to the Korail and Mirpur slums. In Dhaka South City Corporation, 2 Aalo Clinics were also established, which are located adjacent to the Shyampur and Dhalpur slums. Whereas 1 clinic was established in Gazipur City Corporation within the Tongi-Ershadnagar slum and another was established in Narayanganj City Corporation. Urban Health and Demographic Surveillance System (UHDSS) of icddr,b (International Centre for Diarrhoeal Disease Research, Bangladesh), was used as the sampling frame in this study, which includes Korail, Mirpur, Shyampur, Dhalpur, and Tongi-Ershadnagar slums. The UHDSS covers approximately 31,577 households. Details about the UHDSS and its coverage are available elsewhere [10]. However, the inclusion criteria were households covered under UHDSS in the selected slums, household members who had been living with the selected household for at least 6 months before the survey, and households that voluntarily expressed interest in participating in the current study. The exclusion criteria were households not covered by the UHDSS and those not interested in participating in this study.

### Sample Size and Sampling

The household survey served the purpose of assessing the usage of the services of Aalo Clinic to assess the effectiveness of the model in increasing usage of PHC. Concerning this, we aimed to assess the awareness level of the Aalo Clinic model among slum households and expected that approximately 30% of households would be aware of this model and use health care after its implementation. Mahmood et al [12] conducted a study in the same setting and found that 32.2% of sick household members in urban slums used health care from formal health care providers. Considering this proportion, a 5% error level for a 95% CI, and a design effect of 1.2, applied conservatively to account for potential intracluster correlation within slum communities, we estimated a required sample size of 387 for each slum. Furthermore, we considered a nonresponse rate of 10% and projected that the required sample size of 430 households needs to be approached to participate in each study area (total 430×5=2150 households). Finally, we interviewed 2000 households in the baseline survey and 2033 households in the end-line survey. It is important to mention that the sample distribution did not align with the population size of each slum. An equal number of households were sampled from each slum to ensure reliable results. This uniform approach facilitated homogeneity in data collection and supported effective comparative analysis. However, we considered 8919 individuals in the baseline and 7994 individuals in the end-line satisfying the inclusion criteria for chronic disease, with a minimum age of household members 1 year preceding the survey.

A systematic random sampling method was applied to select the households. For each slum, we established an interval between the selected households based on household size and approached them accordingly. For example, approximately 14,000 households reside in the Korail slum and 2300 in the Shyampur slum. For Korail, we used an interval of 32 (14,000/430), and for Shyampur, an interval of 5 (2300/430). If a selected household had multiple eligible members, we randomly chose one to participate in this study using the lottery method. In cases where a household had no eligible member, we proceeded to the next household until we found an eligible participant.

### Data Collection Tool and Variables

We used a pretested and identical semistructured questionnaire to conduct the baseline and end-line surveys. Data were collected through face-to-face interviews with adult household members aged 18 years or older. A total of 12 trained field research assistants were recruited to carry out each survey under the supervision of a field research supervisor. To ensure data quality, this study’s investigators regularly supervised the data collection team and monitored the data to promptly address any issues that arose during the data collection process in this study’s areas. Information on the household members’ demographics, socioeconomic characteristics, illness, health care usage, providers, and knowledge about the Aalo Clinic was collected. Household knowledge, awareness, and usage of the Aalo Clinic were assessed through respondent interviews. Awareness was defined as whether the respondent had ever heard of the Aalo Clinic, while knowledge required correctly identifying it as a local health care facility and naming at least one of its services. Usage referred to any household member receiving services from the Aalo Clinic before the survey, including consultations, disease care, diagnostics, or referrals. Respondents were also asked about their sources of information regarding the Aalo Clinic.

Usually, slum dwellers used health care from drug sellers, quacks, doctors’ chambers, community-based health facilities run by government organizations and NGOs, private clinics and hospitals, government hospitals, medical colleges, and specialized health care institutes [10,12]. In this study, MTPs included doctors with at least an MBBS degree, whether GPs or specialized doctors, who provide health care services through government or nongovernment medical colleges, hospitals, private clinics, or any form of institutional health facilities. These health care organizations hire medically trained staff. In contrast, drug sellers and traditional healers who lack formal training in providing clinical services are categorized as non-MTPs in this study. A similar classification could be found elsewhere [18,21,22]. However, we divided the MTPs based on the Aalo Clinic and other health care facilities that functioned with the MTPs to assess the usage of health care from the MTPs of the Aalo Clinic model.

Evidence reported that in the context of LMICs, the income of informal workers is not stable due to seasonal variations, frequent migration, etc [23,24]. Therefore, to measure the socioeconomic status of slum households, we used asset quintiles rather than income levels. We categorized wealth status into 5 quintiles based on the available assets in the household, such as access to utility services, improved sanitation and drinking water facilities, modern technologies, furniture, etc. These asset variables were used to estimate an asset score through principal component analysis: a robust and validated method widely used in similar contexts. After adjusting for household size, the asset score was used to categorize household wealth status [25]. It is noted that the assessment of wealth quintiles is relative to the current study population and is not comparable to the wealth distribution in the general population of Bangladesh.

### Data Analysis

The background information of the surveyed households, their members, and the health service usage patterns of the sick members are reported as categorical variables with frequency (n) and percentage (%). Out-of-pocket expenditure is presented as the mean and median in Bangladeshi Taka, with US $1=BDT 110. Descriptive analysis was performed to assess distributional changes among surveyed households between the baseline and end-line periods. Additionally, several inferential statistical tests were conducted to assess significant changes between the waves: the Wilcoxon rank sum test for 2 categorical variables, the Kruskal-Wallis test for more than 2 categorical variables, and the *t* test for continuous variables. These ensured the statistical rigor.

Cross-tabulation and chi-squared test were performed to assess the changes in health care usage from MTP among this study’s population between the 2 waves. Further, 3 logistic regression models were applied in assessing the impact of the Aalo Clinic on health care usage from the MTP while controlling for other covariates. This analytic approach enables robust estimation of intervention impact while controlling for potential confounders. In the first model, the binary dependent variable was the “status of health care utilization from the Aalo Clinic” (1=used Aalo Clinic MTP services; 0=did not use Aalo Clinic MTP services). In the second model, the dependent variable was “MTP utilization other than Aalo Clinic” (1=used non–Aalo Clinic MTP services; 0=did not use non–Aalo Clinic MTP services). In the third model, the dependent variable was “status of health care utilization from non-MTPs” (1=used care from non-MTPs; 0=did not use services from non-MTPs). Independent predictor variables included age, sex, marital status, education, occupation, asset quintiles, distance of households, survey type, severity of illness, knowledge, and total health care expenditure. A *P* value of <.05 was considered statistically significant. Data analysis was conducted using Stata SE (version 16.0; StataCorp LLC).

### Ethical Considerations

Ethical approval for this study was obtained from the institutional review board of the icddr,b before data collection (PR#22020). Informed written consent was taken from all the respondents before data collection, after explaining this study’s objectives, the voluntary nature of participation, confidentiality assurances, and their right to withdraw at any time without repercussions. For participants unable to read or write, the consent form was read aloud, and consent was documented via thumb impressions in the presence of an impartial witness. All data were anonymized to ensure privacy and confidentiality, with no personally identifiable information collected or disclosed. Data were stored securely with restricted access to authorized research team members only. No financial or material compensation was provided to participants for their involvement in this study. This paper does not include any images or supplementary materials that could reveal the identity of individual participants.

## Results

### Sociodemographic Characteristics of This Study’s Participants

Both in baseline and end-line surveys, the proportion of males and females was almost equal. At the end-line survey, the proportion of members with no education (n=3047, 38.12%) and secondary and above (n=2485, 31.09%) increased slightly compared to the baseline period. Further, no substantial change was observed in terms of wealth quintiles between the periods. However, there was a sizable increase in the percentage of respondents who knew about Aalo Clinic at the end-line (n=1473, 73.36%) compared to baseline (n=227, 11.46%). Table 1 presents detailed information on the background characteristics of this study’s population.

**Table 1:** Sociodemographic characteristics of study participants at baseline (January to February 2022) and end-line (April to June 2023) from household surveys in the urban slums of Bangladesh.

Variable			Baseline (January to February 2022)		End-line (April to June 2023)
Characteristics of household members			8919 (100)		7994 (100)
**Age (years)**					
	Up to 19		3627 (40.67)		3142 (39.3)
	20-39		3319 (37.21)		3035 (37.97)
	40-59		1475 (16.54)		1383 (17.3)
	60 or more		498 (5.58)		434 (5.43)
**Sex**					
	Male		4460 (50.01)		3957 (49.5)
	Female		4459 (49.99)		4037 (50.5)
**Marital status**					
	Married		4617 (51.77)		4325 (54.1)
	Unmarried		3799 (42.59)		3218 (40.26)
	Others (eg, widows and divorced)		503 (5.64)		451 (5.64)
**Education**					
	No education		3159 (35.42)		3047 (38.12)
	Primary		3009 (33.74)		2462 (30.8)
	Secondary and above		2751 (30.84)		2485 (31.09)
**Occupation**					
	Currently employed		3502 (39.26)		3339 (41.77)
	Currently unemployed		5310 (59.54)		4529 (56.65)
	Others (eg, disabled and retired persons)		107 (1.2)		126 (1.58)
**Regular earner**					
	Yes		3143 (35.24)		3146 (39.35)
	No		5776 (64.76)		4848 (60.65)
Household-level characteristics			1981 (100)		2008 (100)
**Household size**					
	Small (<4)		459 (23.17)		723 (36.01)
	Medium (4-6)		1298 (65.52)		1164 (57.97)
	Large (>6)		224 (11.31)		121 (6.03)
**Household ownership status**					
	Owner		794 (40.08)		700 (34.86)
	Tenant		1187 (59.92)		1308 (65.14)
**Education of the household head**					
	No education		768 (38.77)		751 (37.40)
	Primary		640 (32.31)		636 (31.67)
	Secondary and above		573 (28.92)		621 (30.93)
**Occupation of the household head**					
	Currently employed		1732 (87.43)		1743 (86.80)
	Currently unemployed		214 (10.80)		202 (10.06)
	Others (eg, disabled and retired persons)		35 (1.77)		63 (3.14)
**Asset quintiles**					
	Poorest		423 (21.35)		376 (18.73)
	Poorer		382 (19.28)		416 (20.72)
	Middle		389 (19.64)		408 (20.32)
	Richer		410 (20.7)		388 (19.32)
	Richest		377 (19.03)		420 (20.92)
**City corporation**					
	Dhaka North City Corporation		794 (40.08)		807 (40.19)
	Dhaka South City Corporation		788 (39.78)		798 (39.74)
	Gazipur City Corporation		399 (20.14)		403 (20.07)
**Slum location**					
	Korail		397 (20.04)		404 (20.12)
	Mirpur		397 (20.04)		403 (20.07)
	Shyampur		393 (19.84)		403 (20.07)
	Dholpur		395 (19.94)		395 (19.67)
	Ershadnagar		399 (20.14)		403 (20.07)
**Knowledge about Aalo Clinic**					
	Yes		227 (11.46)		1473 (73.36)
	No		1754 (88.54)		535 (26.64)

### Source of Information About Aalo Clinic

**Figure 2:**
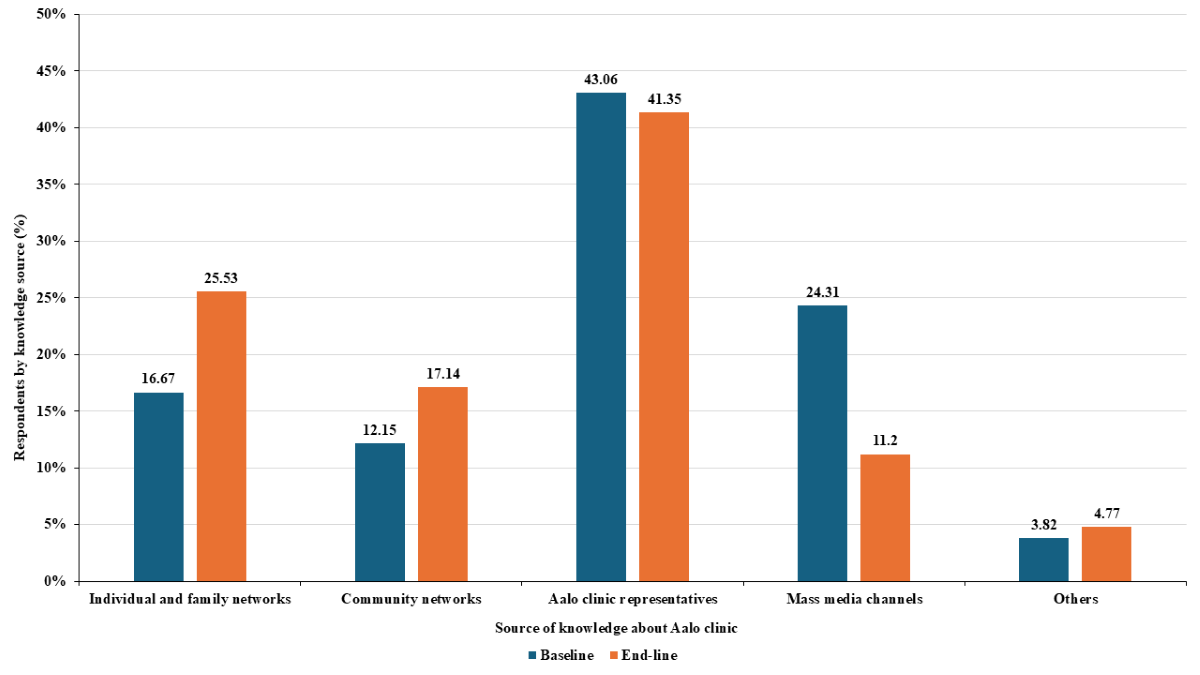
Sources of information about the Aalo Clinic model among urban slum populations in Bangladesh during baseline (January to February 2022) and end-line (April to June 2023) household surveys.

Community networks were the primary source of knowledge about Aalo Clinic in both baseline (n=124, 43.06%) and end-line (n=1030, 41.35%) periods. During the baseline period, mass media channels were the second most common source (n=70, 24.31%). By the end-line, individual and family networks became the second most common source of information (n=636, 25.53%). Further details are presented in Figure 2.

### Chronic Illness and Pattern of Health Care Usage and Expenditure

Around 19% (n=1511) of individuals had a chronic illness at the end-line period, whereas the ratio was about 16% (n=1414) in the baseline period. At the end-line, the highest number of people (n=327, 21.64%) endured chronic musculoskeletal problems, while at the baseline, cardiovascular disease was more common (n=286, 20.23%). At the baseline, around 84% (n=1188) of participants required regular treatment for chronic diseases, but only around 71% (n=1010) received treatment in the past 3 months. At the end-line, around 82% (n=1236) needed regular treatment, but only around 38% (n=566) reported receiving it in the past 3 months. Health care usage from private facilities was significantly lower at the end-line (n=202, 35.69% from n=459, 45.45%), while it was higher for Aalo Clinic (n=63, 11.13% from n=6, 0.59%). However, usage from local drug stores remained almost the same, with 36.63% (n=209) at baseline and 36.93% (n=370) at end-line. Details about the diseases, pattern of health care usage, and expenditures are shown in Table 2.

**Table 2:** Health care usage patterns and expenditures for chronic disease management among urban slum populations in Bangladesh at baseline and end-line surveys.

Variable		Baseline	End-line
**Have a chronic illness or disease, n (%)**		8919 (100)	7994 (100)
	Yes	1414 (15.85)	1511 (18.9)
	No	7505 (84.15)	6483 (81.1)
**Endure a chronic illness for the last 12 months or more, n (%)**			
	Yes	1353 (95.69)	1427 (94.44)
	No	61 (4.31)	84 (5.56)
**Type of chronic illness or disease, n (%)**			
	Heart disease or cardiovascular disease	286 (20.23)	248 (16.41)
	Diabetes	204 (14.43)	231 (15.29)
	Chronic respiratory disease	155 (10.96)	141 (9.33)
	Cancer or tumors	34 (2.40)	40 (2.65)
	Hepatobiliary problems	29 (2.05)	31 (2.05)
	Neurological problem or disease	107 (7.57)	97 (6.42)
	Chronic eye problem	87 (6.15)	109 (7.21)
	Chronic gastrointestinal problems	100 (7.07)	82 (5.43)
	Skin problem	46 (3.25)	108 (7.15)
	Chronic musculoskeletal problems	251 (17.75)	327 (21.64)
	Chronic urinary disorder or problem	64 (4.53)	32 (2.12)
	Others (eg, anemia, sexual, etc)	51 (3.61)	65 (4.30)
**Daily performance decreased due to the chronic illness, n (%)**			
	Yes	1126 (79.63)	1151 (76.17)
	No	288 (20.37)	360 (23.83)
**Need regular treatment for chronic illness, n (%)**			
	Yes	1188 (84.02)	1236 (81.8)
	No	226 (15.98)	275 (18.2)
**Received treatment for the chronic illness in the last 3 months, n (%)**			
	Yes	1010 (71.43)	566 (37.46)
	No	404 (28.57)	945 (62.54)
**Type of health care provider for chronic illness, n (%)**			
	Aalo Clinic provider	6 (0.59)	63 (11.13)
	Medically trained providers (non–Aalo Clinic)	616 (60.99)	285 (50.35)
	Nonmedically trained providers	388 (38.42)	218 (38.52)
**Source of treatment for chronic illness, n (%)**			
	Public facility	148 (14.65)	73 (12.9)
	Private facility	459 (45.45)	202 (35.69)
	Aalo Clinic	6 (0.59)	63 (11.13)
	NGO^a^ facility	9 (0.89)	10 (1.77)
	Pharmacy or local drug store	370 (36.63)	209 (36.93)
	Others	18 (1.78)	9 (1.59)
**Designation of the service providers for chronic illness, n (%)**			
	Qualified doctor (allopathic)	603 (59.7)	342 (60.42)
	Qualified doctor (homeopathic, unani, or ayurvedic)	13 (1.29)	7 (1.24)
	Unqualified doctor (homeopathic, unani, or ayurvedic)	10 (0.99)	10 (1.77)
	Drug seller	366 (36.24)	203 (35.87)
	Others (eg, traditional healer)	18 (1.78)	4 (0.71)
**Distance of the facility from respondents’ home (min, baseline=798, end-line=484), n (%)**			
	Nearby areas (within 15)	291 (36.47)	173 (35.74)
	Moderately distant areas (16 to 30)	214 (26.82)	129 (26.65)
	Distant areas (more than 30)	293 (36.72)	182 (37.6)
**Waiting time for health care services for chronic illness (min, baseline=798, end-line=484), n (%)**			
	Within 10	392 (49.12)	202 (41.74)
	11-20	124 (15.54)	47 (9.71)
	More than 20	282 (35.34)	235 (48.55)
**Total expenditure for health care seeking for chronic illness**			
	Mean (SD)	2608 (7330)	2365 (6654)
	Median (IQR)	1200 (2190)	980 (2020)
**Total expenditure of health care seeking by health care facility type for chronic illness, mean (SD)**			
	Public facility	3427 (8457)	2476 (3629)
	Private facility	3924 (9346)	4535 (10,457)
	Aalo Clinic	590 (809)	708 (601)
	NGO facility	1165 (970)	362 (495)
	Pharmacy or local drug store	691 (866)	850 (756)
	Others	867 (903)	1336 (1896)
**Total expenditure of health care seeking by health care facility type for chronic illness, median (IQR)**			
	Public facility	1635 (1880)	1510 (2150)
	Private facility	2140 (3120)	2760 (3560)
	Aalo Clinic	400 (310)	550 (570)
	NGO facility	1500 (1550)	100 (546)
	Pharmacy or local drug store	400 (510)	630 (500)
	Others	500 (770)	800 (780)

^a^NGO: nongovernmental organization.

### Health Care Seeking Behavior

**Figure 3:**
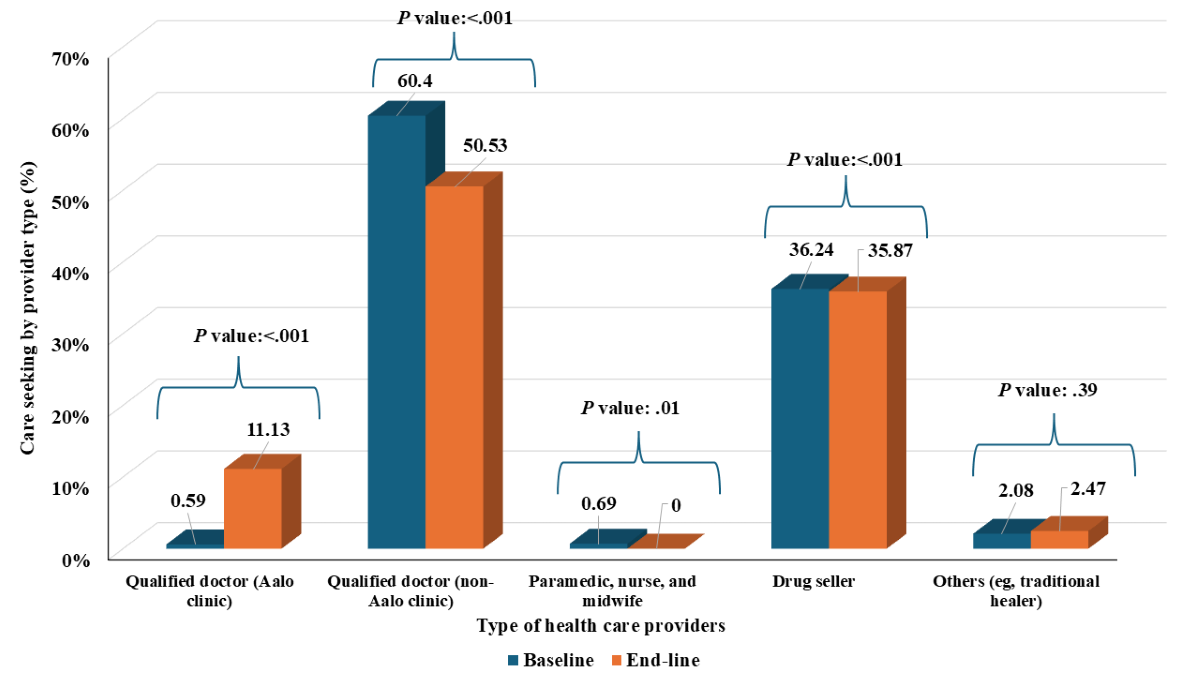
Health care–seeking behavior for chronic diseases by type of health care providers among urban slum populations in Bangladesh during baseline and end-line surveys.

Figure 3 presents the preference of health care providers for chronic disease among slum dwellers. Health care usage from MTP at Aalo Clinic was significantly higher, rising from 0.59% (n=6) in the baseline period to 11.13% (n=63) in the end-line period. In both periods, non–Aalo Clinics and local drug sellers were the main health care sources, with non–Aalo Clinics at 60.40% (n=610) in the baseline and 50.53% (n=285) in the end-line, and local drug sellers at 36.24% (n=366) in the baseline and 35.87% (n=206) in the end-line. Reliance on non-MTPs declined slightly, and although this reduction was statistically significant, the magnitude of change was minimal.

### Health Care Usage of Chronic Disease Across Various Health Care Providers

At baseline, female patients (n=6, 0.98%), married individuals (n=4, 0.53%), those with no education (n=5, 0.97%), household heads with secondary education or higher (n=3, 0.97%), the unemployed (n=5, 1.33%), irregular earners (n=6, 0.97%), individuals from the richest households (n=3, 1.02%), and those who experienced a reduction in working performance due to chronic illness (n=5, 0.60%) predominantly used health care services from Aalo Clinic compared to their counterparts. A similar pattern was observed at the end-line period for the usage of Aalo Clinic MTPs. Females (n=54, 13.64%), widows or divorced individuals (n=14, 12.96%), household heads with no education (n=26, 11.82%), the unemployed (n=43, 15.69%), currently employed household heads (n=43, 9.45%), irregular earners (n=51, 15.6%), those in the richest asset quintile (n=19, 10.73%), and those who experienced a reduction in working performance due to chronic illness (n=59, 12.72%) also predominantly used health care services from Aalo Clinic compared to their counterparts. Table 3 presents a detailed distribution of patients’ health care usage across various providers between 2 periods.

**Table 3:** Distribution of patients with chronic disease by type of health care providers used at baseline and end-line surveys in urban slums of Bangladesh.

Variable			Baseline			End-line		
			Aalo Clinic provider	Medically trained providers (non–Aalo Clinic)	Nonmedically trained providers	Aalo Clinic provider	Medically trained providers (non–Aalo Clinic)	Nonmedically trained providers
**Characteristics of household members, n (%)**								
	**Age (years)^a^**							
		Up to 19	0 (0)	46 (74.19)	16 (25.81)	2 (4.55)	26 (59.09)	16 (36.36)
		20-39	2 (0.65)	202 (65.16)	106 (34.19)	23 (11.79)	96 (49.23)	76 (38.97)
		40-59	3 (0.75)	226 (56.22)	173 (43.03)	26 (11.87)	113 (51.60)	80 (36.53)
		60 or more	1 (0.42)	142 (60.17)	93 (39.41)	12 (11.11)	52 (48.15)	44 (40.74)
	**Sex^b^**							
		Male	0 (0)	255 (64.39)	141 (35.61)	9 (5.29)	88 (51.76)	73 (42.94)
		Female	6 (0.98)	361 (58.79)	247 (40.23)	54 (13.64)	197 (49.75)	145 (36.62)
	**Marital status^c^**							
		Married	4 (0.53)	453 (60.48)	292 (38.99)	46 (11.19)	207 (50.36)	158 (38.44)
		Unmarried	0 (0)	58 (77.33)	17 (22.67)	3 (6.38)	28 (59.57)	16 (34.04)
		Others (eg, widows and divorced)	2 (1.08)	105 (56.45)	79 (42.47)	14 (12.96)	50 (46.3)	44 (40.74)
	**Education^d^**							
		No education	5 (0.97)	298 (57.98)	211 (41.05)	36 (13.38)	129 (47.96)	104 (38.66)
		Primary	1 (0.38)	151 (56.77)	114 (42.86)	17 (11.26)	76 (50.33)	58 (38.41)
		Secondary and above	0 (0)	167 (72.61)	63 (27.39)	10 (6.85)	80 (54.79)	56 (38.36)
	**Education of the household head^e^**							
		No education	1 (0.24)	239 (56.64)	182 (43.13)	26 (11.82)	106 (48.18)	88 (40)
		Primary	2 (0.72)	161 (57.71)	116 (41.58)	22 (13.5)	79 (48.47)	62 (38.04)
		Secondary and above	3 (0.97)	216 (69.9)	90 (29.13)	15 (8.2)	100 (54.64)	68 (37.16)
	**Occupation^f^**							
		Currently employed	0 (0)	236 (57.7)	173 (42.3)	13 (5.2)	121 (48.4)	116 (46.4)
		Currently unemployed	5 (1.33)	220 (58.51)	151 (40.16)	43 (15.69)	137 (50)	94 (34.31)
		Others (eg, disabled and retired persons)	1 (0.44)	160 (71.11)	64 (28.44)	7 (16.67)	27 (64.29)	8 (19.05)
	**Occupation of the household head^g^**							
		Currently employed	5 (0.61)	491 (60.25)	319 (39.14)	43 (9.45)	231 (50.77)	181 (39.78)
		Currently unemployed	0 (0)	45 (56.96)	34 (43.04)	12 (19.67)	24 (39.34)	25 (40.98)
		Others (eg, disabled and retired persons)	1 (0.86)	80 (68.97)	35 (30.17)	8 (16)	30 (60)	12 (24)
	**Regular earner^h^**							
		Yes	0 (0)	231 (58.93)	161 (41.07)	12 (5.02)	119 (49.79)	108 (45.19)
		No	6 (0.97)	385 (62.3)	227 (36.73)	51 (15.6)	166 (50.76)	110 (33.64)
	**Household size^i^**							
		Small (<4)	1 (0.79)	80 (63.49)	45 (35.71)	18 (11.76)	81 (52.94)	54 (35.29)
		Medium (4-6)	3 (0.43)	416 (60.29)	271 (39.28)	37 (10.76)	163 (47.38)	144 (41.86)
		Large (>6)	2 (1.03)	120 (61.86)	72 (37.11)	8 (11.59)	41 (59.42)	20 (28.99)
	**Asset quintiles^j^**							
		Poorest	1 (0.7)	87 (60.84)	55 (38.46)	5 (6.41)	27 (34.62)	46 (58.97)
		Poorer	0 (0)	82 (56.94)	62 (43.06)	7 (8.24)	44 (51.76)	34 (40)
		Middle	1 (0.49)	114 (55.61)	90 (43.9)	15 (13.27)	49 (43.36)	49 (43.36)
		Richer	1 (0.44)	140 (62.22)	84 (37.33)	17 (15.04)	61 (53.98)	35 (30.97)
		Richest	3 (1.02)	193 (65.87)	97 (33.11)	19 (10.73)	104 (58.76)	54 (30.51)
	**Ownership of a living house^k^**							
		Owner	5 (0.86)	363 (62.59)	212 (36.55)	37 (12.63)	154 (52.56)	102 (34.81)
		Tenant	1 (0.23)	253 (58.84)	176 (40.93)	26 (9.52)	131 (47.99)	116 (42.49)
	**City corporation^l^**							
		Dhaka North City Corporation	1 (0.26)	233 (61.15)	147 (38.58)	18 (7.86)	116 (50.66)	95 (41.48)
		Dhaka South City Corporation	0 (0)	243 (61.83)	150 (38.17)	20 (11.76)	92 (54.12)	58 (34.12)
		Gazipur City Corporation	5 (2.12)	140 (59.32)	91 (38.56)	25 (14.97)	77 (46.11)	65 (38.92)
	**Study area (slums)^m^**							
		Korail	0 (0)	137 (68.5)	63 (31.5)	7 (5.83)	73 (60.83)	40 (33.33)
		Mirpur	1 (0.55)	96 (53.04)	84 (46.41)	11 (10.09)	43 (39.45)	55 (50.46)
		Shyampur	0 (0)	122 (68.16)	57 (31.84)	7 (8.64)	39 (48.15)	35 (43.21)
		Dholpur	0 (0)	121 (56.54)	93 (43.46)	13 (14.61)	53 (59.55)	23 (25.84)
		Ershadnagar	5 (2.12)	140 (59.32)	91 (38.56)	25 (14.97)	77 (46.11)	65 (38.92)
**Type of chronic illness or disease, n (%)^n^**								
	Heart disease or cardiovascular disease		2 (0.9)	112 (50.68)	107 (48.42)	9 (10.59)	37 (43.53)	39 (45.88)
	Diabetes		2 (1.16)	101 (58.38)	70 (40.46)	16 (14.04)	53 (46.49)	45 (39.47)
	Chronic respiratory disease		0 (0)	73 (60.83)	47 (39.17)	4 (7.84)	23 (45.1)	24 (47.06)
**Total expenditure of health care seeking by health care provider type for chronic illness^r^**								
	Mean (SD)		590 (809)	3760 (9065)	698 (867)	708 (601)	3855 (9066)	863 (802)
	Median (IQR)		400 (310)	2000 (2870)	400 (540)	550 (570)	2295 (3630)	630 (500)

### Econometric Analysis

Factors associated with health care usage for chronic disease from MTPs of Aalo Clinic, non–Aalo Clinic, and non-MTPs are shown in Table 4. Female household members were over 3 times more likely (odds ratio [OR] 3.68, 95% CI 1.54-8.79) to use services from MTPs at Aalo Clinic compared to their male counterparts. Furthermore, individuals residing within the nearby areas were 2 times more likely (OR 2.77, 95% CI 1.08-7.11), and individuals residing within a moderate distance from Aalo Clinic were 3 times more likely (OR 3.23, 95% CI 1.23-8.48) to use its services compared to those living at a longer distance. Compared to the baseline period, household members were 23 times more likely (OR 23.28, 95% CI 8.77-61.80) to use MTPs at Aalo Clinic during the end-line period. Patients whose daily performance decreased due to chronic illnesses were 3 times more likely (OR 3.27, 95% CI 1.16-9.23) to take services from MTPs at Aalo Clinic during the end-line period. Additionally, household members who had knowledge about Aalo Clinic were twice as likely (OR 2.13, 95% CI 1.09-4.14) to use its MTPs compared to those without such knowledge.

**Table 4:** Factors associated with health care usage from Aalo Clinic, non–Aalo Clinic, medically trained and non-MTPs^a^ for chronic disease management among urban slum populations in Bangladesh.

Characteristics of household members		Aalo Clinic provider, OR^b^ (95% CI)	Medically trained providers (non–Aalo Clinic), OR (95% CI)	Non-MTPs, OR (95% CI)
**Age (years)**				
	Up to 19	Reference	Reference	Reference
	20-39	1.44 (0.12-17.42)	1.06 (0.25-4.47)	0.77 (0.20-2.96)
	40-59	1.85 (0.14-24.57)	0.83 (0.19-3.63)	0.90 (0.23-3.63)
	60 or more	2.00 (0.14-28.76)	1.26 (0.27-5.81)	0.66 (0.16-2.76)
**Sex**				
	Male	Reference	Reference	Reference
	Female	3.68^c^ (1.54-8.79)	0.91 (0.62-1.33)	0.85 (0.59-1.22)
**Marital status**				
	Married	Reference	Reference	Reference
	Unmarried	1.43 (0.17-12.29)	2.35 (0.60-9.21)	0.40 (0.11-1.43)
	Others (eg, widows and divorced)	0.69 (0.30-1.59)	0.76 (0.47-1.23)	1.37 (0.87-2.15)
**Education of the household head**				
	No education	0.81 (0.40-1.65)	1.25 (0.84-1.87)	0.83 (0.57-1.21)
	Primary	Reference	Reference	Reference
	Secondary and above	0.66 (0.30-1.45)	2.05^c^ (1.33-3.16)	0.58^c^ (0.38-0.87)
**Occupation of the household head**				
	Currently employed	0.92 (0.40-2.11)	0.92 (0.57-1.49)	1.12 (0.71-1.78)
	Currently unemployed	Reference	Reference	Reference
	Others (eg, disabled and retired persons)	2.51 (0.62-10.13)	1.48 (0.54-4.05)	0.44 (0.16-1.19)
**Distance of the facility from respondents’ home**				
	Nearby areas	2.77^d^ (1.08-7.11)	0.04^e^ (0.02-0.06)	23.15^e^ (13.92-38.47)
	Moderately distant areas	3.23^c^ (1.23-8.48)	0.09^e^ (0.06-0.15)	10.23^e^ (6.11-17.12)
	Distant areas	Reference	Reference	Reference
**Household size**				
	Small (<4)	0.67 (0.22-2.00)	1.55 (0.83-2.87)	0.67 (0.37-1.23)
	Medium (4-6)	0.62 (0.23-1.64)	1.01 (0.62-1.65)	1.01 (0.63-1.63)
	Large (>6)	Reference	Reference	Reference
**Asset quintiles**				
	Poorest	0.56 (0.19-1.66)	1.19 (0.68-2.09)	0.99 (0.59-1.68)
	Poorer	0.68 (0.24-1.96)	1.21 (0.70-2.11)	0.96 (0.57-1.60)
	Middle	Reference	Reference	Reference
	Richer	1.38 (0.58-3.26)	1.34 (0.82-2.21)	0.73 (0.45-1.16)
	Richest	1.04 (0.44-2.49)	1.49 (0.91-2.45)	0.73 (0.46-1.17)
**Study area (slums)**				
	Korail	Reference	Reference	Reference
	Mirpur	2.47 (0.76-8.02)	0.31^e^ (0.18-0.54)	2.29^c^ (1.38-3.79)
	Shyampur	2.44 (0.68-8.76)	0.37^c^ (0.21-0.66)	2.17^c^ (1.25-3.76)
	Dholpur	2.47 (0.75-8.09)	0.59^d^ (0.35-1.00)	1.27 (0.77-2.10)
	Ershadnagar	4.03^c^ (1.36-11.91)	0.63 (0.38-1.03)	1.12 (0.70-1.79)
**Survey type**				
	Baseline	Reference	Reference	Reference
	End-line	23.28^e^ (8.77-61.80)	0.33^e^ (0.23-0.48)	1.22 (0.87-1.72)
**Daily performance decreased due to chronic illness**				
	Yes	3.27^c^ (1.16-9.23)	2.13^c^ (1.36-3.36)	0.39^e^ (0.25-0.60)
	No	Reference	Reference	Reference
**Knowledge about Aalo Clinic**				
	Yes	2.13^c^ (1.09-4.14)	1.03 (0.61-1.75)	0.68 (0.41-1.10)
	No	Reference	Reference	Reference
Total expenditure for the chronic illness		0.9991^e^ (0.9986-0.9996)	1.0008^e^ (1.0006-1.0010)	0.9993^c^ (0.9992-0.9995)

^a^MTP: medically trained provider.

^b^OR: odds ratio.

^c^*P*<.01.

^d^*P*<.05.

^e^*P*<.001.

On the other hand, patients were 2 times more likely (OR 2.05, 95% CI 1.33-3.16) to use non–Aalo Clinic MTPs if their household head had a secondary or higher education level compared to those whose household head had only a primary education level. Individuals residing nearby or within a moderate distance from non–Aalo Clinic MTPs were less likely to use these services compared to those living at a longer distance. Individuals from the richest asset quintiles were about 1.5 times more likely (OR 1.49, 95% CI 0.91-2.45) to use non–Aalo Clinic MTPs compared to those in the middle quintile of this study’s population. During the end-line period, household members were less likely (OR 0.33, 95% CI 0.23-0.48) to use non–Aalo Clinic MTPs compared to the baseline period. Moreover, patients whose daily performance decreased due to chronic illnesses were 2 times more likely (OR 2.13, 95% CI 1.36-3.36) to take services from MTPs at non–Aalo Clinics during the end-line period.

Further, we found that households where the household head had an education of secondary or above level were less likely (OR 0.58, 95% CI 0.38-0.87) to use non-MTPs compared to others. Individuals residing nearby and within a moderate distance from non-MTPs were about 23 times and 10 times more likely (OR 23.15, 95% CI 13.92-38.47 and OR 10.23, 95% CI 6.11-17.12) to use these services compared to those living at a longer distance, respectively. Members' richest asset quintiles were less likely (OR 0.73, 95% CI 0.45-1.16) to use non-MTPs compared to those in the middle quintile. Additionally, household members who possessed knowledge about Aalo Clinic were less likely (OR 0.68, 95% CI 0.41-1.10) to use non-MTPs compared to those without such knowledge. However, the association was not statistically significant.

## Discussion

### Principal Results and Comparison With Prior Work

The pilot Aalo Clinic model offered an essential package of health services, including chronic diseases, and an opportunity for outpatient PHC for the urban low-income population.

We found that health care use from Aalo Clinic MTPs for chronic diseases was significantly associated with higher use compared to other MTPs. The Aalo Clinic’s unique features, such as the availability of affordable and quality health care services for chronic diseases, may significantly influence its impact on the community. The multivariate logistic regression analysis demonstrated a significant effect that individuals were over 23 times more likely to use Aalo Clinic MTP services at end-line than baseline. A similar initiative, namely the “Mohalla Clinic” in Delhi, India, has been implemented [26], and its impact aligns with the findings of this study to make primary care accessible and affordable from MTP for urban underserved populations [27,28]. However, despite these positive trends, the intervention did not substantially reduce the usage of health care from non-MTPs. Non-MTP providers remain prevalent and widely trusted within these communities. The continued reliance on non-MTPs in slum areas can be attributed to several factors, including deeply rooted health care–seeking behaviors, cultural preferences, limited medicine supply, and the convenience and accessibility of informal providers such as drug sellers and traditional healers. Shifting patient preferences away from non-MTPs is a complex and gradual process that requires sustained engagement.

Knowledgeable individuals can make informed decisions when choosing health care providers [29]. The integration of awareness measures in our analysis is a strength, offering a deeper understanding of demand-side dynamics. Among the slum population, knowledge about Aalo Clinic increased significantly, which may be associated with greater usage of health care services from Aalo Clinic MTPs compared to other MTPs. A study conducted by Hasan et al [21] identified factors that influence health care usage from the MTP of a government-sponsored health protection scheme in Bangladesh. The scheme covers the population who are below the poverty line and live in semiurban and rural areas. However, this study reported that households with knowledge about the scheme were significantly associated with higher health care usage from the MTP of that scheme.

While evidence from LMICs often shows higher health care usage among males [30-32], our study found that females were more likely to access Aalo Clinic MTP services. Several contextual factors may explain this finding. The strategic placement of Aalo Clinics within slum areas enhances accessibility, while the provision of privacy, reduced costs, and shorter travel distances are particularly important for women in the urban poor settings of Bangladesh. These factors likely contribute to the higher uptake of services among females at Aalo Clinics compared to other MTPs. This observation is consistent with findings from studies among similar and general populations in Bangladesh [33,34], highlighting the importance of placing clinics close to communities to reduce sex-based access barriers.

This study identified a noteworthy pattern in health care usage, demonstrating a significant association between the distance from households and the type of health care provider. Our findings indicate that individuals are more motivated to seek care from providers located in closer proximity, irrespective of whether the provider is an MTP or a non-MTP. This observation aligns with previous studies, which have similarly highlighted the critical role of proximity in influencing health care–seeking behavior [35,36]. On the other hand, the significant positive association between longer distance and higher health care usage, specifically from non–Aalo Clinic MTPs, reveals an interesting trend. This could be indicative of several factors, such as specialization of health facilities for specific services, reputation, and the referral system [37]. Non–Aalo Clinic MTPs provide advanced laboratory tests, specialist consultations, and inpatient care unavailable at the more basic primary care level of Aalo Clinics. This may be associated with patients traveling from farther away or being referred to non–Aalo Clinic MTPs for services not offered locally, resulting in higher usage of these more distant providers. Hasan et al [21] also found that, though health care, medicine, and diagnostic services are free in a government-sponsored health protection scheme, a higher proportion of people still sought care from other MTPs located within a similar or greater distance.

Though there was no significant difference in health care usage from Aalo Clinic MTPs across asset quintiles, a significant difference was observed in the usage of health care services from non–Aalo Clinic MTPs and non-MTPs across asset quintiles. Patients who use health care from non–Aalo Clinic MTPs where those who belong to the richest quintiles were more likely to use health care from non–Aalo Clinic MTPs; whereas those who use health care from non-MTPs, among those who belong to the richest quintiles, were less likely to seek health care from non-MTPs. Ahmed et al [18] conducted a study in Bangladesh and showed a similar finding that individuals of the richest households are more likely to use health care than individuals of the poorest households. Moreover, evidence reported that people who belong to the richest quintiles are less likely to use health care from non-MTPs [38,39].

Nevertheless, the Aalo Clinic model has been delivering free-of-cost essential health care services for chronic diseases for the low-income slum population, a substantial portion of them opt for informal providers, such as drug sellers. This behavior may be rooted in long-standing historical and cultural patterns of health care seeking within these communities. Traditionally, many slum residents have relied on informal providers such as drug sellers, traditional healers, or unlicensed practitioners due to their easy accessibility, flexible hours, and lower immediate costs. These providers are often trusted members of the community who offer quick remedies, sometimes with payment options that formal providers cannot match. Additionally, limited awareness of the benefits of MTPs, coupled with barriers such as distance, cost, and social norms, has reinforced preferences for informal care.

Consequently, changing these entrenched behaviors will require sustained time and concerted efforts. The widespread presence of informal providers, combined with the lower travel time and out-of-pocket costs associated with accessing them, further perpetuates their use. To effectively address this challenge, it is crucial to establish and maintain satellite clinic services within the community over an extended period. These clinics can serve as accessible platforms for educating slum residents on the importance of regularly using qualified MTPs for chronic disease management, while minimizing travel and financial barriers.

Both affordability and quality of health care are critical components of UHC. Thus, it is important to emphasize these. Though Aalo Clinic has made notable advancements in enhancing access to quality PHC, concerns regarding the affordability of its services remain, primarily due to associated costs. Despite the clinic’s strategic location for slum dwellers and its provision of free registration, consultations, essential medicines, and diagnostic or laboratory services, users may still incur significant health care expenses or face higher opportunity costs. These financial burdens may often arise from the limited availability of certain medicines and laboratory services at Aalo Clinics, necessitating that patients seek these resources externally, thereby increasing out-of-pocket payments. To mitigate this affordability issue, it is imperative to ensure that Aalo Clinics are adequately stocked with essential medicines and laboratory services. Close collaboration with government stakeholders responsible for urban PHC provision should be pursued to address these gaps effectively. Enhancing the operational readiness of the model could help reduce out-of-pocket expenses and improve equitable access to affordable health care services, thereby advancing progress toward UHC.

Even though the Aalo Clinic model is associated with higher health care usage, it faces a significant challenge in the form of extended waiting times for accessing services. This issue likely arises from either an influx of patients or an inadequate number of health care providers. Each Aalo Clinic center is staffed with only 1 male and 1 female GP per shift, which may prove insufficient given the patient load. Research indicates that longer waiting times are often negatively correlated with perceived quality of care [40]. To address this issue effectively, it is crucial to improve both the infrastructure and human resource capacity of the Aalo Clinic. Enhancing these aspects could help mitigate waiting times and improve overall patient satisfaction and service delivery.

### Limitations

One of the key limitations is that we were unable to account for seasonal variations in health care usage because the baseline survey was conducted from January to February 2022, while the end-line survey occurred between April and June 2023. Additionally, our reliance on self-reported data introduces the possibility of recall bias. Furthermore, the repeated cross-sectional design of this study limits our ability to make causal inferences about the relationship between significant factors and health care usage. Therefore, to better understand these associations over time, future research using a longitudinal design is warranted. As we did not follow the same households over time, it is difficult to determine whether observed changes are directly attributable to the Aalo Clinic intervention or influenced by external factors, such as other NGOs, government schemes, or broader contextual changes in the urban health system. Finally, the use of equal sample sizes across slums, regardless of population size, may limit generalizability, although it allowed for better cross-site comparison.

### Conclusions

The Aalo Clinic model is significantly associated with increased usage of MTPs for chronic disease care, with patients showing a shift in preference from other MTPs, particularly private providers, to Aalo Clinics. Although there was a statistically significant but modest reduction in reliance on non-MTPs, informal providers continue to serve as an important source of care in low-income, underserved slum areas. To support continuity of care for chronic diseases requiring regular treatment, Aalo Clinic authorities should maintain consistent availability of essential medications. Future longitudinal research is warranted to better understand the drivers of patient preferences for chronic diseases and the longer-term impact of the Aalo Clinic model before scale-up, providing evidence to inform strategies for strengthening urban PHC and advancing progress toward UHC for low-income slum populations in Bangladesh.

## Data Availability

The datasets used and/or analyzed during this study are available from the corresponding author upon reasonable request.

## Abbreviations

GP: general practitioner
icddr,b: International Centre for Diarrhoeal Disease Research, Bangladesh
LMIC: low- and middle-income country
MTP: medically trained provider
NGO: nongovernmental organization
OR: odds ratio
PHC: primary health care
UHC: universal health coverage
UHDSS: Urban Health and Demographic Surveillance System
